# Genetic risk of Klinefelter's syndrome in assisted reproductive technology

**DOI:** 10.1002/rmb2.12029

**Published:** 2017-04-04

**Authors:** Tamito Miki, Motoi Nagayoshi, Yoichi Takemoto, Takashi Yamaguchi, Satoru Takeda, Seiji Watanabe, Atsushi Tanaka

**Affiliations:** ^1^ Saint Mother Obstetrics and Gynecology Clinic and Institute for ART Kitakyushu Japan; ^2^ Department of Obstetrics and Gynecology Juntendo University School of Medicine Tokyo Japan; ^3^ Department of Anatomical Science Hirosaki University Graduate School of Medicine Hirosaki Japan

**Keywords:** fluorescent in situ hybridization, intracytoplasmic sperm injection, Klinefelter's syndrome, microsurgical testicular sperm extraction, X‐chromosome short tandem repeat analysis

## Abstract

**Aim:**

The main cause of Klinefelter's syndrome (KS) has been believed to be XY sperm. Accordingly, in the intracytoplasmic sperm injection treatment of patients with KS, hereditary KS has been a concern. Therefore, this study attempted to estimate the risk before and after the assisted reproductive technology.

**Methods:**

First, in order to validate the safety of the gametes of the patients with KS, a fluorescent in situ hybridization (FISH) analysis, following an original cell identification method using 1052 testicular gametes of 30 patients, was conducted. Second, in the resultant 45 babies, cytogenetic and physical–cognitive screening data were analyzed. In addition, a first attempt was conducted to investigate the origin of the extra X chromosome in 11 patients with KS by using 12 X‐chromosome short tandem repeat (STR) analysis in order to estimate the paternal contribution to KS.

**Results:**

No sex chromosomally abnormal gamete was found in the FISH analysis and the babies were normal genetically, physically, and cognitively. In the STR, it was confirmed that most (7/11) of the patients with KS resulted from the fertilization of the XX oocytes, suggesting that a baby with KS that had been reported previously might not have resulted from XY sperm.

**Conclusion:**

These results indicate that the risk of assisted reproductive technology for patients with KS is not as high as previously expected.

## 1 Introduction

Klinefelter's syndrome (KS) is one of the most common chromosomal abnormalities that are found in newborns. Its incidence is ~1 in 500‐1000 live births in men.^1^ Almost all the patients with KS have a non‐mosaic 47 XXY karyotype,^2^ originally described as a syndrome with increased‐exertion follicle‐stimulating hormone (FSH), gynecomastia, azoospermia,^3^, ^4^ and a slightly low IQ. Once considered to be permanently infertile, these individuals can now reproduce using intracytoplasmic sperm injection (ICSI) of spermatozoa that are extracted from their testicles.^5^ To date, the birth of >200 babies has been reported around the world^6^, ^7^, ^8^, ^9^, ^10^, ^11^, ^12^, ^13^, ^14^, ^15^, ^16^, ^17^, ^18^, ^19^, ^20^, ^21^, ^22^, ^23^ and 45 of those babies were born at the Saint Mother Obstetrics and Gynecology Clinic and Institute for ART, Kitakyushu, Japan, following ICSI with the gametes of patients with KS. Among those cases, the authors know only of one case of a fetus in a triplet pregnancy that was diagnosed with KS with amniocentesis; that case was later reduced to a healthy twin pregnancy.^10^ In contrast, there have been reports warning that the sex chromosome abnormality incidence increases in children who are born as a result of ICSI from patients with KS who produce sperm with XY disomy.^24^, ^25^, ^26^, ^27^, ^28^, ^29^ However, other reports^12^ showed that only normal sperm with haploid X or Y were found in the testis of patients with KS. Accordingly, it is important to collect more precise data in order to determine the risk level of assisted reproductive technology (ART) by using the gamete of a patient with KS and in order to decide on how to deal with the clinical treatment of patients with KS.

Recently, the authors established criteria to distinguish testicular somatic and meiotic cells without fixation and any staining,^30^ and accordingly, the precise cytogenetic analysis of these cells became possible. In this study, the criteria for a cytogenetic analysis (chromosome assay and fluorescent in situ hybridization [FISH]) were used so that the sperm and meiotic cells of patients with KS would be selectively isolated, instead of the previous method with a spermatogenic cell mixture. Furthermore, a follow‐up review of a comparably large number (n=45) of newborn babies that were born from the ART treatment of patients with KS at the institute from 2000 to 2013 was performed in order to confirm that XY sperm were not selected for the ART treatment. Namely, the risk of hereditary KS was examined before and after the ART treatment. In addition, a first attempt of X‐chromosome short tandem repeat (STR) analysis was conducted among patients and their parents in order to estimate XY sperm and XX oocyte contribution to the birth of patients with KS, as there is a possibility that KS resulting from XX oocyte fertilization might be more frequent.

## 2 Materials and Methods

### 2.1 Patients

This study dealt with 280 men who had been diagnosed previously as having non‐mosaic KS and had consented to receive microsurgical testicular sperm extraction (micro‐TESE) treatment at the institute from 2000 to 2013.

### 2.2 Biopsy of the testis tissues

Several different sites of each testis were biopsied under an operation microscope (micro‐TESE). The biopsied testicular tissues were prepared in Dulbecco's phosphate‐buffered saline (DPBS) containing 0.125% collagenase and 0.01% DNase in order to free the spermatogenic cells from the seminiferous tubules, as previously described.^30^ The cells were used for cytogenetic analysis or clinical treatments (ICSI or round spermatid injection [ROSI]) after cryopreservation.

### 2.3 Freezing and thawing of the sperm or spermatid

For the cryopreservation of the sperm, the testis tissue suspension was centrifuged and the pellets were transferred into a droplet of human tubal fluid (HTF) on a Petri dish with a glass pipette under a diverted microscope. Then, the cell suspension was mixed with a very small amount of a freezing medium (~2 μL of HTF with 10% serum protein substitute [SPS; Origio, Malov, Denmark] and 100 mmol/L sucrose) and placed on the tip of Cryotop (Kitazato Corp., Fuji, Japan) under an inverted microscope. The Cryotop was exposed to liquid nitrogen vapor for 2 minutes and stored in liquid nitrogen. For the thawing of the frozen cells, after maintaining the Cryotop in air for 5 seconds, it was dipped into a droplet that was covered with warm mineral oil (37°C) to suspend the cells.^31^ The motile sperm were selected and used for ICSI.

For ROSI, the spermatids were selected from the testicular cell suspension under an inverted microscope and suspended in 0.15 mL of freezing medium (DPBS with 0.6 mol/L ethylene glycol, 0.125 mol/L sucrose, and antibiotics). The suspension was drawn in a 0.25 mL Cassou straw and cooled on ice (4°C). After the straw was maintained at −7°C in a cooling chamber of a programmable alcohol bath freezer for 20 minutes, it was cooled to −30°C at the rate of −0.3°C/min before being plunged into liquid nitrogen. Thawing was carried out by maintaining the straw in air for 5 seconds. The cell suspension then was diluted with HTF containing 10% SPS in a test tube to remove the cryoprotectant.^30^

### 2.4 Cytological identification of the testicular cells

The authors already have established the criteria for identifying biopsied spermatogenic cells morphologically. The characteristics of the testicular cells were examined in detail under a differential interference microscope (10×40) and then their chromosomal constitution was determined by cytogenetic analysis to confirm whether the characteristics used and the meiotic phases correlated with each other.^30^

### 2.5 Fluorescent in situ hybridization procedure

Spermatogonia (SG), primary spermatocytes (Pr‐Scs), round spermatids (STs), and elongating or mature sperm that had been selected from enzymatically treated biopsied tissues with a micromanipulator were put into a droplet of HTF with SPS and then placed in a droplet of 10 μL of PBS (C^2+^‐ and Mg^2+^‐free) on Poly‐L‐Lysine (Sigma‐Aldrich, St Louis, MO, USA)‐coated slides with a square mark on the back slide. Soon after the PBS was placed, the surrounding cells were completely dried off and covered with a small amount of the fixative of Carnoy's solution (methanol: acetic acid=3:1). The fixative evaporated gradually and the cells became transparent. When the fixative completely disappeared, the cell membrane burst and the nuclei were attached to the glass slide. In order to wash away the phosphate crystals that were derived from the PBS (–), the cells were covered with the fixative several times and dried in air. For the fixed cells, triple‐target FISH was performed by using fluorescence‐labeled DNA probes that were specific for chromosomes X and Y and chromosome 7 (CEP DNA probe; Vysis, Downers Grove, IL, USA). The mixture of the probes was applied to the slide under a cover slip and the nuclear and probe DNA were denatured simultaneously for 5 minutes at 75°C. The slide then was incubated in a chamber (Hybrite; Vysis) at 42°C for 120 minutes in order to allow hybridization, followed by counterstaining with 4,6‐diamidino‐2‐pheniylindole (Vector Laboratories, Inc., Burlingame, CA, USA).^32^

### 2.6 Chromosome assay of the spermatogenic cells

For the chromosome analysis of the spermatogenic cells that were identified with the authors’ morphological criteria, a chromosome assay with interspecific injection into mouse MII oocytes was used.^33^ The meiotic cells were injected into the mouse oocytes 10 min after electrical stimulation (8 V/cm AC, 1000 KHz for 8 seconds, and 1200V/cm DC for 99 μseconds). Sperm or elongating sperm were injected without electric stimulation. After overnight incubation in the HTF containing 50 ng/mL vinblastine, the nucleus of the meiotic cells or injected sperm was allowed to form chromosomes in the mouse oocytes. Chromosome slides of the oocytes were prepared by the gradual fixation‐air drying method.^34^

### 2.7 Microinjection of the testicular sperm or spermatid

Intracytoplasmic sperm injection was conducted with motile and morphologically normal sperm. Oocyte penetration by the spermatid was conducted with a comparably larger injection pipette than that used for conventional ICSI. In the cases of the STs and STs with a small flagellum, oocyte activation with electrical stimulation was required before injection,^30^ but no oocyte activation was required for the elongating–elongated spermatids. After 5 days of incubation, the blastocysts were transferred into the uterus.

### 2.8 X‐chromosome short tandem repeat analysis

Using blood or oral mucous samples of 11 patients with KS and one or both of their parents with their consent, the origin of the extra X chromosome was determined with X‐chromosome haplotype markers (STRs of 12 loci), according to the method of one particular study.^35^ With DNA extracted from the samples, multiplexed polymerase chain reaction (PCR) amplifications of the 12 X‐STR loci (linkage group 1: DXS10148, DXS10135, DXS8378; linkage group 2: DXA10079, DXS10074, DXS7132; linkage group 3: HPRTB, DXS10101, DXS10103; linkage group 4: DXS10134, DXS10146, DXS10146) and amelogenin were conducted by using an Investigator Argus X‐12 QS Kit (Qiagen, Hilden, Germany). Electrophoresis was run on an ABI PRISM 3100 Genetic Analyzer (Applied Biosystems, Foster City, CA, USA) for the PCR products. The obtained data were analyzed with GeneMapper ID software (Thermo Fisher Scientific Inc., Waltham, MA, USA). All the steps described above were entrusted to Tohoku Chemical Company, Ltd., Tohoku, Japan.

### 2.9 Screening of babies and children

Out of the 45 babies who were born by ICSI or spermatid injection treatment from the patients with KS in the institute, 29 underwent chromosomal analysis by using amniocentesis or peripheral blood samples before or after birth, respectively, along with the compulsory newborn or infant screening for physical and cognitive development in Japan. For the rest, only information in the screening was used to examine the possibility of KS.

### 2.10 Ethical considerations

The clinical application of ROSI and genetic analysis with X‐chromosome STRs were approved by the Institutional Review Board of the Saint Mother Obstetrics and Gynecology Clinic for ART, Kitakyushu, Japan. This clinical study also was registered on the University Hospital Medical Information Network of Japan (UMIN Clinical Trials Registry: UMIN000006117, UMIN000024542), adhering to the Recommendations for the Conduct, Reporting, Editing, and Publication of Scholarly Work in Medical Journals criteria.

## 3 Results

### 3.1 Morphological characteristics of the spermatogenic cells

**Figure 1:**
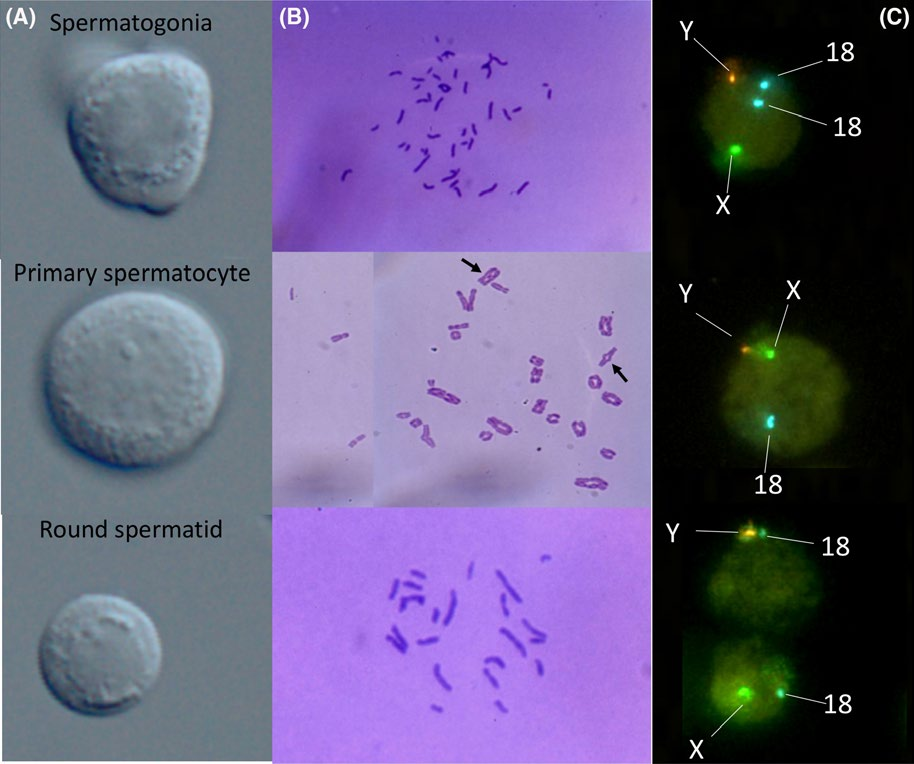
Morphology and chromosomal constitution of a normal spermatogonium, primary spermatocyte, or round spermatid. The three types of spermatogenic cells that were identified under a differential interference microscope were: A, their chromosome complements formed in the mouse oocytes; B, fluorescent signals of X and Y; and C, 18 chromosomes by fluorescent in situ hybridization analysis

In Figure 1A, typical images of the spermatogenic cells are shown. The elongating and elongated spermatids were easily identified, with deviated condensed nuclei and short flagella, respectively. It was comparably difficult to distinguish among the SG, early Pr‐Scs, and STs. The STs were the smallest spermatogenic cells (6‐8 μm in diameter; slightly smaller than erythrocytes). They were much smaller than the Pr‐Scs (10‐12 μm) and slightly smaller than the SG (8‐10 μm). Two‐to‐three nucleoli were seen within the nuclei of the SG and Pr‐Scs, but not in the STs. An area of the cytoplasm surrounding the nucleus was narrower in the STs than in the SG. Protruded active pseudopodia often were seen in the SG,^36^ but not in the STs. Although an acrosomal vesicle or cap was considered to be strong evidence of the cell being a ST, such structures were found in <10% of the presumptive spermatids.

### 3.2 Chromosome abnormality in the testicular cells of the patients with Klinefelter syndrome

In Figures 1B and C, chromosomal images of normal spermatogenic cells that were visualized by chromosomal assay and FISH analysis, respectively, are shown. When the chromosomes of the spermatogenic cells were induced to condense in the mouse oocytes, the SG had 46 of the dyad chromosomes, which are seen at the metaphase of somatic cell proliferation. The Pr‐Scs had 23 of the tetrad chromosomes, in some of which the cross‐overs were observed (see the arrow in Figure 1B). The STs had 23 monad chromosomes. Therefore, the chromosome assay showed that the authors’ criteria for spermatogenic cell morphology allowed the cells to be identified correctly, and accordingly, the authors could apply the cells that were selected with the criteria for a FISH analysis of their interphase nuclei. In the SG, two blue spots of chromosome 18 and a green and orange spot of X and Y were found. In the Pr‐Scs, each one spot of chromosome 18, X, and Y were visible, three spots in total. In the STs, a blue spot of chromosome 18 and either a green (X) or orange (Y) spot were visible, a total of two spots (Figure 1C).

The results of the FISH analysis for the SG, Pr‐Scs, and STs in five men with KS are shown in Table 1. The mean values of the men's age, testicular volume, FSH, luteinizing hormone, and testosterone were: 33.2 years old, 11.2 mL, 4.08 mIU/mL, 4.32 mIU/mL, and 4.48 ng/mL, respectively. In the SG stage, the average proportion of the 46 XY and 47 XXY chromosomes was ~73.6% (194/265) and 26.4% (71/265), respectively. In one case (no. 2), all the SG were of 46 XY chromosomal constitution (34/34). Until now, there have been few reports describing the high percentage of 46 XY, compared to 47 XXY, chromosomes. This result deserves attention. In contrast, in all five men with KS, no sex chromosomally aberrant Pr‐Sc was found (0/256) and the resultant STs (467 cells) also were normal with haploid X or Y in almost equal proportion (49:51). This result seems to strongly support the probability that chromosomally normal sperm and STs are derived from the meiosis of chromosomally normal Pr‐Scs in patients with KS. In addition, in the other 25 patients with KS, the 100 sperm (n=10) or 485 STs (n=15) that were selected were subjected to a FISH analysis in order to estimate the risk of accidentally selecting gametes with sex chromosome aneuploidy for ICSI. In conclusion, no sex chromosome abnormalities have been observed in the gametes of patients with KS until now.

**Table 1:** Fluorescent in situ hybridization analysis of the spermatogenic cells from five patients with Klinefelter's syndrome

Patient	Spermatogonia		Primary spermatocyte		Spermatid		
	XY (%)	XXY (%)	XY (%)	XXY (%)	X (%)	Y (%)	Other (%)
1	79 (95.2)	4 (4.8)	106 (100.0)	0 (.0)	132 (49.8)	133 (50.2)	0
2	34 (100.0)	0 (.0)	31 (100.0)	0 (.0)	15 (41.7)	21 (58.3)	0
3	21 (45.7)	25 (54.3)	36 (100.0)	0 (.0)	24 (48.0)	26 (52.0)	0
4	33 (57.9)	24 (42)	49 (100.0)	0 (.0)	27 (49.1)	28 (50.9)	0
5	27 (60.0)	18 (40.0)	34 (100.0)	0 (.0)	31 (50.8)	30 (49.2)	0
Total	194 (73.6)	71 (26.4)	256 (100.0)	0 (.0)	229 (49.0)	238 (51.0)	0

### 3.3 Success rate of intracytoplasmic sperm injection and round spermatid injection

The success rates of the ICSI and ROSI treatment of patients with KS are shown in Table 2. Out of 280 patients, sperm with faint motility were recovered in 92 (32.9%) patients for ICSI. Spermatids, which are evidence of the completion of meiosis, were found in 33 (11.8%) patients for ROSI. In 155 (55.4%) patients, no spermatogenic cell was found. The incidence of pregnancy per treatment cycle, miscarriage, and delivery were 12.4% (59/477), 37.3% (22/59), and 7.8% (37/477), respectively. Finally, 45 healthy babies (including six twins and one triplet case) were born (male: female= 21:24).

**Table 2:** Clinical outcome of micro‐fertilization using the gamete of patients with Klinefelter's syndrome

Variable	Sperm	Spermatid (Sa, Sb)	Spermatid (Sc, Sd)
No. of patients	92 (32.9%)	8 (2.9%)	25 (8.9%)
Age of wife (years)	31.2 (25‐36)	30.5 (24‐34)	29.5 (23‐33)
No. of collected oocytes/patient	12.4 (8‐20)	11.4 (7‐19)	10.4 (6‐15)
No. of fertilized oocytes/patient	8.1 (6‐15)	7.9 (5‐13)	7.5 (5‐11)
% of good Day 3 embryos	60.4% (4.9/8.1)	30.3% (2.4/7.9)	44.0% (3.3/7.5)
Implantation rate^a^	17.4% (51/293)	14.5% (9/62)	16.4% (20/122)
Pregnancy rate^a^	13.7% (40/293)	9.7% (6/62)	10.7% (13/122)
Miscarriage rate^a^	32.5% (13/40)	66.7% (4/6)	38.5% (5/13)
Delivery rate^a^	9.2% (27/293)	3.2% (2/62)	6.6% (8/122)

a Per embryo transfer cycle. Forty‐five babies were delivered in 37 cases that included six twin and one triplet pregnancies. Sa, round spermatid; Sb, round spermatid with a small flagellum; Sc, elongating spermatid; Sd, elongated spermatid.

### 3.4 Physical and cognitive development of the babies of patients with Klinefelter syndrome

In the 29 babies who were cytogenetically analyzed, it was confirmed that they had a normal karyotype. The results of the newborn or infant screening for physical and cognitive development also showed that, in all 45 babies, no abnormality was found.

### 3.5 Origin of the extra X chromosome in patients with Klinefelter syndrome

Examples of X‐chromosomal STR DNA profiles are shown in Table 3. Patient no. 09KY is a case in which both X chromosomes were inherited from the mother (maternal origin). The allele of the DXS10148 locus was 22.1 in the patient, 20 in his father, and 20, 22.1 in his mother, suggesting that the patient's allele was inherited from the maternal X chromosomes. In the other loci, all the alleles of the patient were consistent with those of the mother. In the patient case of paternal origin (22TK), all the alleles of the 12 X‐chromosome loci were inherited from the father, indicating that the patient's X chromosomes came from both the father and the mother. In 63.6% (seven) of the patients who were examined, the X chromosome was inherited from the mother and in 36.4% (four) of the 11 cases, it was inherited from the father. In the patients who had two maternal origin X chromosomes, the cause of KS is that an extra X chromosome was left in an oocyte as a result of chromosomal non‐disjunction at the first or second meiotic division. In the patient who had X chromosomes inherited from both parents, fertilization of the XY sperm is the cause of KS.

**Table 3:** Examples of X‐chromosome short tandem repeat profiles of patients with Klinefelter's syndrome and their parents

Patient no.	Marker	Father	Patient	Mother
Maternal origin of extra X chromosome				
09KY	DXS10148	*20*	**22.1**	20, **22.1**
	DXS10135	*22*	**22**	21, **22**
	DXS8378	*10*	**10**	**10**
	DXS10079	*18*,* 21*	**18**,** 20**	17, **18**,** 20**
	DXS10074	*17*	**18**	16, **18**
	DXS7132	*16*	**14**	**14**, 15
	HPRTB	*12*	**14**	13, **14**
	DXS10101	*31.2*	**29**,** 31.2**	**29**,** 31.2**
	DXS10103	*18*	**17**,** 19**	**17**,** 19**
	DXS10134	*35*	**36**,** 37.3**	**36**,** 37.3**
	DXS10146	*24*,* 40.2*	**26**,** 32**	**26**,** 32**
	DXS7423	*14*	**15**,** 16**	**15**,** 16**
	AM	*X*,* Y*	X, *Y*	X
Paternal origin of the extra X chromosome				
22TK	DXS10148	*25.1*	**24.1**,* 25.1*,** 28.1**	24.1, 27.1, **28.1**
	DXS10135	*26*	**19**,* 26*	**19**, 31
	DXS8378	*12*	**11**,* 12*	**11**
	DXS10079	*16*	**15**,* 16*,** 21**	**15**, 19, **21**
	DXS10074	*18*	**17**,* 18*	**17**, 19
	DXS7132	*14*	**14**,* 14*	13, **14**
	HPRTB	*13*	**12**,* 13*,** 14**	**12**,** 13**,** 14**
	DXS10101	*31*	*31*,** 31.2**	**31.2**, 32.2
	DXS10103	*19*	**18**,* 19*	**18**
	DXS10134	*34*	*34*,** 36**	**36**
	DXS10146	*28*	**26**,* 28*	**26**
	DXS7423	*14*	*14*,** 16**	15, **16**
	AM	*X*,* Y*	X, *Y*	X

**Bold**, allele that patients inherited from the mother; *italics*, paternal allele.

## 4 Discussion

### 4.1 Genetic risk of the intracytoplasmic sperm injection treatment for patients with Klinefelter syndrome

The present study showed that 45 babies were successfully delivered by using oocyte penetration by sperm or spermatid from patients with KS from January 2000 to December 2013 at the institute and, among them, there was neither a case of chromosomal abnormality nor any case of physical or cognitive abnormality. The miscarriage rate (37.3%) in the treatment of patients with KS by using sperm and spermatid was not significantly higher, when compared with patients who do not have KS (20.1% of 134).^30^ The results indicate the possibility that the genetic risk of the embryos that are produced in the treatment of patients with KS is not as high as previously believed.

This clinical result is consistent with the cytogenetic data of the FISH and chromosomal analysis in the gametes from patients with KS. In the 25 patients with KS who were examined, no sex chromosome abnormality was found in 952 ST cells and 100 sperm (Table 2). The mechanism to produce normal gametes in the testis of patients with KS is considered to be as follows. In patient no. 2, all the SG and Pr‐SCs that were analyzed were XY in their sex chromosome constitution. Therefore, there is no doubt that STs with X or Y chromosomes could be derived from the meiosis of sex chromosomally normal germ cells. In the remaining four patients with KS with testicular mosaicism of XY and XXY SG, it is difficult to determine which of the XY or XXY cells were the source of the STs. However, in all of their Pr‐SCs that were analyzed, the sex chromosome constitution was XY, and accordingly, all the ST cells might have been produced from XY SG, suggesting the possibility that XXY SG cannot enter meiosis. Another study also has reported that there was no XXY pachytene gamete and no increase of XY STs or XY sperm in three testicular 46 XY/47XXY mosaic patients with KS, reaching the conclusion that 46 XY cells can undergo meiosis.^12^ There is another possibility that the resultant abnormal daughter cells of XXY SG can become degenerative or apoptotic,^37^ because in this study, only the spermatogenic cells that were alive with the intact plasma membrane and smooth round shape were selectively examined. This possibility seems to be a reason for an inconsistency of the present data with those of previous cytogenetic studies in patients with KS. Many previous FISH studies have reported that not only the sex chromosome abnormality rate, but also the rate of autosomal aneuploidies,^24^ is higher in sperm from patients with KS than from infertile patients without KS.^24^, ^25^, ^26^, ^27^, ^28^, ^29^ In those studies, the testicular cell suspension was directly smeared onto a glass slide, treated with dithiothreitol, and hybridized with FISH probes. As after the successive treatment, the artificially swollen sperm heads were not allowed to be evaluated for their morphology, a tail was used to identify the sperm. Therefore, it cannot be denied that aberrant sperm heads, which are not appropriate for ICSI treatment, must have been analyzed along with normal sperm heads in the previous FISH studies. It is a clear fact that the risks of disomy and diploidy are higher in sperm with aberrant heads.^38^, ^39^ In addition, this assumption is supported by the high frequency of XY sperm that has been found in the control donor sperm that are used in FISH studies because the rates of XY sperm that were obtained were 20‐100‐fold higher than the rate (0.018%) that was reported by a study that used a chromosome assay of 15 864 ejaculated donor sperm (n=51) that penetrated hamster oocytes.^40^ The authors understand that the reason for the distinct results between the current and the previous FISH studies cannot be revealed without a comparative study among the different sperm selection procedures. However, it can be concluded that, instead of using a testicular cell suspension, the authors’ cytogenetic studies with spermatogenic cells that were morphologically evaluated and selected are more suitable for the exact estimation of the genetic risk in the ICSI treatment of patients with KS. In constrast, a study reported an increase of sex chromosome aneuploidy in array comparative genomic hybridization with the trophoblasts that had been biopsied from embryos obtained by the ICSI treatment of men with oligozoospermia,^41^ suggesting the risk of the use of suboptimal sperm. Although it is not clear whether patients with KS are included in their data, the result seems to disagree with the present data. However, their data include some points that are hard to understand. First, the total aneuploidy rates did not differ among the embryos from IVF and ICSI with normal and suboptimal sperm groups. Second, in the embryos of the suboptimal sperm group, aneuploidy increased in specific autosomes in addition to the sex chromosomes. These incompatible phenomena seem to be explained by the possibility that patients with genetic backgrounds causing aneuploidy of a specific chromosome(s) are contained in the oligozoospermia group. Therefore, their result might not necessarily be applicable to patients with KS, although close attention has to be paid to the genetic risk of ICSI treatment of patients with KS.

### 4.2 Contribution of the XX oocyte in the production of Klinefelter syndrome

When the current study found that no XY aneuploidy could be observed in the gametes of patients with KS in the authors’ cytogenetic analysis, it was hypothesized that the XY sperm did not contribute to the production of KS as much as the XX oocytes. In this X‐chromosome STR analysis, the patients with maternal origin X chromosomes were comparably frequent (63.6%), suggesting that the contribution of the XX oocyte to the production of XXY embryos might be greater than by the XY sperm, although the sample number that was applied for the X‐chromosomal STR DNA profiling is not large enough. Some studies have previously attempted to determine the origin of the extra X chromosome in patients with KS with X‐chromosome restriction site polymorphism.^42^, ^43^, ^44^ The maternal contribution to the production of KS was slightly greater in two studies (59% vs 41%) and was slightly lower in one study (42.8% vs 57.1%). In those studies, however, there were cases in which the X‐chromosome origin was determined by the appearance or disappearance of a single band in a single allele, which might have resulted from mutation. In the X‐chromosome STR analysis, the 12 X‐chromosomal markers are clustered into four linkage groups, which consist of three alleles, and thus each set of three markers is handled as a haplotype for genotyping to avoid misjudgment. The authors could find no previous study that applied X‐chromosome STR with PCR to patients with KS. One study reported that the extra X chromosome is the result of meiotic non‐disjunction^45^ or possibly, as recently described, of the premature separation of sister chromatids, both paternally or maternally, because of an increased maternal age.^1^, ^46^ As the X‐chromosome origin can affect the potency of spermatogenesis in patients with KS, the authors will collect further data by using this method.

## Disclosures

*Conflict of interest*: The authors declare no conflict of interest. *Human and Animal Rights*: All the procedures were followed in accordance with the ethical standards of the responsible committees on human experimentation (institutional and national) and with the Helsinki Declaration of 1964 and its later amendments. Informed consent was obtained from all the patients to be included in the study. All the institutional and national guidelines for the care and use of laboratory animals were followed.
